# Maternal obesity during lactation as a hypertensive cardiovascular risk factor in the offspring

**DOI:** 10.1113/EP093824

**Published:** 2026-07-16

**Authors:** Gabriela Arenas, Cristián A. Amador, Susana Contreras‐Duarte

**Affiliations:** ^1^ Facultad de Ciencias Universidad San Sebastián Santiago Chile; ^2^ Facultad de Ciencias de la Rehabilitación y Calidad de Vida Universidad San Sebastián Santiago Chile

**Keywords:** breast milk, cardiovascular programming, hypertension, maternal obesity

## Abstract

Maternal obesity is a major public health concern, with growing evidence supporting its role in the developmental programming of cardiometabolic disease. Although prenatal mechanisms have been investigated extensively, the contribution of lactation as a postnatal window of cardiovascular (CV) programming remains insufficiently understood. Breast milk is a complex and dynamic biofluid that contains immune mediators, hormones, lipids and extracellular vesicles, which actively shape early‐life immune maturation, metabolic regulation and vascular development. Emerging data indicate that maternal obesity is associated with alterations in breast milk immune mediators, adipokines and lipid composition, particularly an increased omega‐6 to omega‐3 polyunsaturated fatty acid ratio, and extracellular vesicle‐associated microRNAs. These changes might influence endothelial function, oxidative stress balance, inflammatory signalling pathways, neurohumoral regulation and adipose tissue development in the offspring during a critical postnatal window of CV maturation. This review synthesizes current evidence on how maternal obesity reshapes the immunological, microbiological, hormonal, lipidic and epigenetic landscape of breast milk and discusses the potential implications for offspring hypertensive CV programming. We highlight key knowledge gaps, including the need for comprehensive characterization of maternal metabolic status, longitudinal vascular assessment in offspring and mechanistic studies that distinguish between pre‐ and postnatal influences. Understanding how lactation‐mediated signals contribute to early vascular priming might offer new opportunities for preventive strategies to reduce intergenerational transmission of CV risk.

## Abstract (highlights)

- **What is the topic of this review?**
  This review focuses on lactation as a critical postnatal window, during which maternal obesity might influence cardiovascular development and long‐term cardiovascular risk in the offspring.
- **What advances does it highlight?**
  It integrates evidence indicating that maternal obesity during lactation is associated with postnatal exposures that can modulate vascular, renal, metabolic and neuroendocrine pathways involved in cardiovascular homeostasis. The review highlights lactation as an underexplored period of cardiovascular programming, distinct from gestational influences, and identifies key knowledge gaps regarding the mechanisms linking maternal obesity during lactation to offspring cardiovascular risk.

## 1 INTRODUCTION

Obesity, defined as body mass index (BMI) ≥ 30 kg/m^2^, has emerged as a major global health concern, with prevalence increasing consistently among women of reproductive age (Poston et al., 2016; World Health Organization, 2023). Globally, the prevalence of maternal obesity is estimated at 20.9% and is projected to reach 23.3% by 2030 (Kent et al., 2024). Approximately one‐third of these women remain obese during the postpartum period, when lactation occurs (Spencer et al., 2015), and the evidence indicates that offspring exposed to maternal obesity during early life are at increased risk of adverse outcomes, including obesity, hypertension and dyslipidaemia, among others (Hochner et al., 2012). Collectively, these conditions represent well‐established risk factors for the development of cardiovascular (CV) disease later in life, which remains a leading cause of mortality worldwide, accounting for ∼17.9 million deaths annually (Kajikawa & Higashi, 2022; World Health Organization, 2024).

Consistent with the concept of developmental origins of health and disease, adverse environmental exposures during critical periods of early development increase susceptibility to CV disease in adulthood (Lacagnina, 2020). Several biological mechanisms have been proposed to underlie the programming effects of maternal obesity on offspring cardiometabolic risk, including alterations in appetite regulation, physical activity, muscle development and adipocyte biology (Jahan‐Mihan et al., 2025). However, most studies have focused on maternal obesity during pregnancy or on combined exposure during pregnancy and lactation, whereas the specific contribution of the lactation period remains comparatively underexplored. Recent evidence highlights lactation as a critical postnatal window that can shape offspring development and long‐term health, as the infant continues to depend on maternal metabolic status through breast milk exposure (Picó et al., 2021).

**FIGURE 1:**
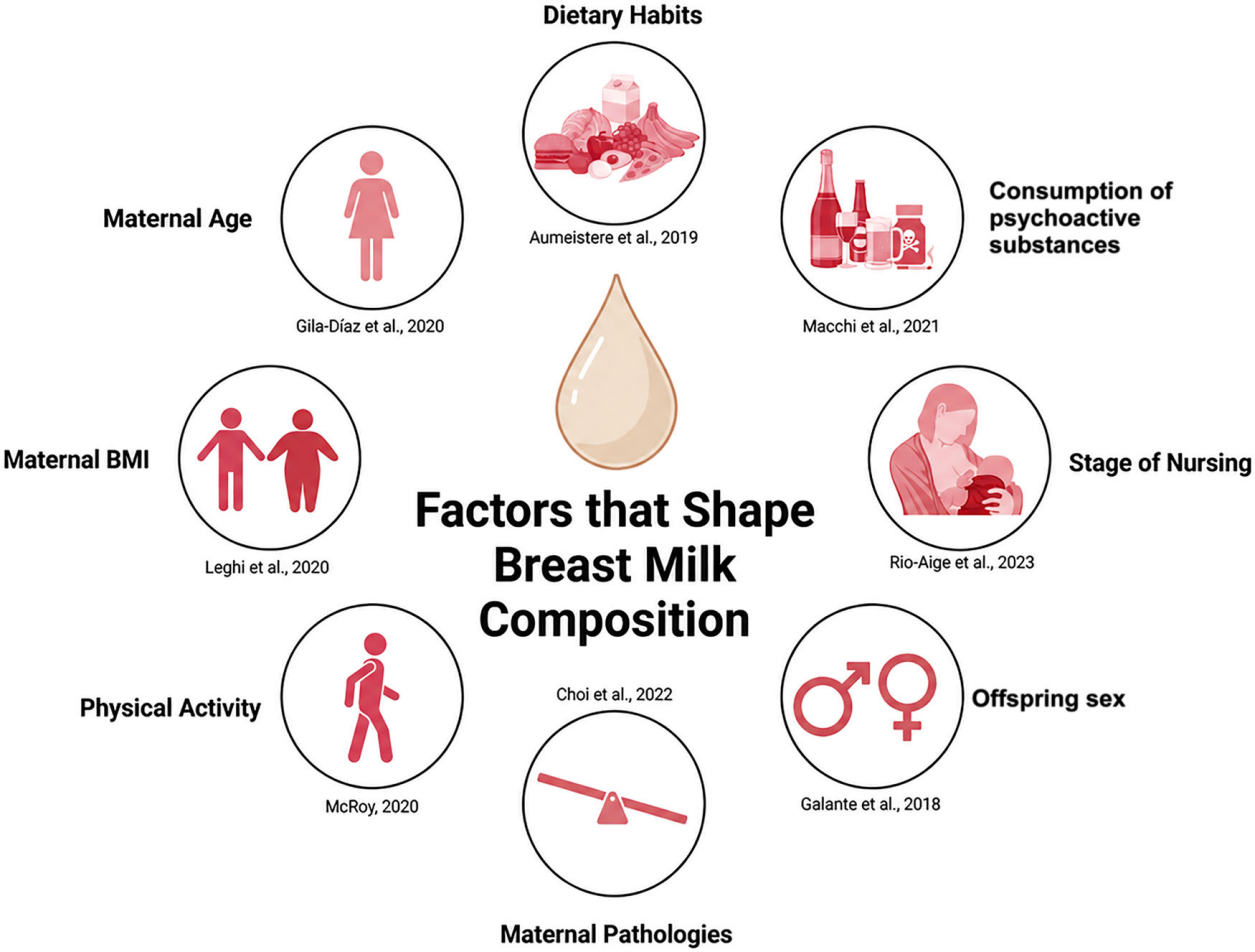
Interaction of maternal, lifestyle, environmental and biological factors in shaping breast milk composition during lactation. Multiple factors, including maternal BMI, dietary patterns, lifestyle behaviours, environmental exposures and characteristics of the mother–infant dyad, can interact to influence the nutritional and bioactive composition of breast milk. Image created with BioRender.com. Abbreviation: BMI, body mass index.

Breast milk composition reflects the integration of multiple maternal lifestyle and biological factors, including diet, metabolic and inflammatory status, environmental exposures and characteristics of the mother–infant dyad, that collectively shape its nutritional and bioactive profile (Aumeistere et al., 2019; Choi et al., 2022; Galante et al., 2018; Gila‐Díaz et al., 2020; Leghi et al., 2020; Macchi et al., 2021; McRoy, 2020; Rio‐Aige et al., 2023; Figure 1). In this context, current evidence suggests that certain nutritional components of breast milk might differ according to offspring sex. Some studies have reported higher energy content in milk produced for male offspring; however, findings remain heterogeneous across populations, and a clear male or female predominance has not been established (Galante et al., 2018). Additionally, maternal obesity has been associated with alterations in the nutritional and bioactive composition of breast milk, including changes in lipid and fatty acid profiles, hormones and adipokines, inflammatory cytokines/chemokines and molecular cargos, such as extracellular vesicles (EVs) and microRNAs (miRs). These maternal obesity‐associated modifications might act as physiological signals that influence early‐life pathways involved in the infant metabolic regulation, vascular function and CV development (Arenas et al., 2025). Accordingly, the aim of this review is to summarize and critically discuss current evidence on the impact of maternal obesity on breast milk composition and its potential role in offspring CV programming. We propose that obesity‐induced alterations in breast milk converge on biological pathways that shape vascular, metabolic and renal outcomes during early postnatal life, providing a mechanistic link between lactation and long‐term hypertensive CV risk.

## 2 PHYSIOLOGICAL ROLE OF BREAST MILK IN POSTNATAL DEVELOPMENT

Breast milk is widely recognized as the optimal source of nutrition for newborns and infants, providing a unique combination of nutrients and bioactive factors that support growth, development and immune protection during early life (Kalarikkal & Pfleghaar, 2023; World Health Organization, 2023). According to the World Health Organization, it is recommended to breastfeed exclusively for the first 6 months of life to maximize its health benefits (World Health Organization, 2023). In physiological conditions, breastfeeding is associated with a reduced incidence of major infectious diseases, such as diarrhoea and pneumonia, and there is a lower prevalence of chronic conditions later in life, including obesity and diabetes, in breastfed infants (Hossain & Mihrshahi, 2022).

Breast milk is a complex biofluid composed predominantly of water (∼87%–88%), with carbohydrates, lipids and proteins accounting for most of its solid fraction (Kim & Yi, 2020). In addition to its nutritional constituents, breast milk contains a wide range of bioactive and immunological components, including enzymes, hormones, growth factors, immunoglobulins (Igs), cytokines, chemokines, immune cells and EVs, which contribute to its functional properties (Andreas et al., 2015; Eisha et al., 2022). Some milk constituents are synthesized locally by mammary secretory cells, whereas others are transferred from the maternal circulation, highlighting the close physiological link between maternal metabolic status and milk composition (Burgoyne & Duncan, 1998; Shennan & Peaker, 2000). Collectively, these components have evolved to provide immune protection and regulate metabolic homeostasis, appetite control, and tissue and organ development during early life in the offspring (Bermejo‐Haro et al., 2023). However, these components are sensitive to maternal physiological status and can be altered in conditions such as maternal obesity, potentially modifying the biological signals transmitted to the offspring during this critical developmental window.

## 3 MATERNAL OBESITY AS A STATE OF NEUROHORMONAL AND INFLAMMATORY DYSREGULATION DURING LACTATION

Against this physiological background, maternal obesity represents a state of chronic metabolic and inflammatory dysregulation that extends into the lactation period, perturbing systemic maternal physiology. Beyond its role as a metabolic tissue, the mammary gland constitutes a specialized and highly responsive organ, whose function during lactation depends on the tight integration of epithelial–stromal interactions and tightly regulated endocrine, paracrine and neurohormonal signalling pathways (Zhou et al., 2025). In physiological conditions, infant suckling activates neuroendocrine reflex pathways that stimulate hypothalamic and pituitary signalling involved in prolactin and oxytocin release (Gomez‐Casado et al., 2025). These hormones are essential for coordinating milk synthesis and milk ejection from the mammary gland (Gomez‐Casado et al., 2025). In mothers with obesity, experimental and clinical evidence indicates that increased circulating inflammatory mediators, insulin resistance and altered adipokine signalling can disrupt hypothalamic–pituitary pathways and impair mammary gland responsiveness to prolactin and oxytocin (De Los Ríos et al., 2018; Luzardo‐Ocampo et al., 2023). This dysregulation can interfere with the normal activation of dopaminergic and neuroendocrine circuits that gate prolactin secretion and with oxytocinergic pathways that coordinate myoepithelial contraction, thereby blunting the coordinated hormonal response to suckling (Kamikawa & Seko, 2020; Luzardo‐Ocampo et al., 2023; Silva et al., 2020). Consequently, obese mothers exhibit diminished prolactin and oxytocin responses to suckling during lactation, which might contribute to impaired milk synthesis and defective milk ejection reflexes (Kopelman, 2000; Rasmussen & Kjolhede, 2004). These features have been associated with delayed onset of lactogenesis, reduced milk production and shortened duration of lactation, contributing to premature breastfeeding cessation (Luzardo‐Ocampo et al., 2023; Marshall et al., 2019; Turcksin et al., 2014). Collectively, these alterations reflect a broader state of neurohormonal and inflammatory dysregulation associated with maternal obesity that persists into the lactation period and might influence breast milk composition, with potential implications for offspring development.

## 4 POSSIBLE BREAST MILK‐MEDIATED MECHANISMS LINKING MATERNAL OBESITY DURING LACTATION TO OFFSPRING HYPERTENSIVE CARDIOVASCULAR RISK

The early postnatal period represents a critical phase of CV, metabolic and immune maturation, during which lactation constitutes the primary postnatal interface linking maternal physiology to the developing offspring (Amaro et al., 2022; Khan et al., 2023). During early postnatal life, the immature intestinal barrier facilitates the transfer of bioactive milk‐derived signals into the systemic circulation of the offspring, thereby increasing the potential for maternally derived factors to influence developing physiological systems (Bankole & Li, 2025; Weström et al., 2020). As a result, breastfed infants of mothers with obesity might be exposed to a distinct profile of bioactive milk components capable of modulating pathways involved in immune maturation, metabolic regulation, vascular function and CV development (Amaro et al., 2022). However, the extent to which these alterations can be attributed specifically to maternal obesity remains difficult to determine in human studies. Many studies group women with overweight, obesity, excessive gestational weight gain (EGWG) or broader metabolic disturbances, including gestational diabetes and hypertensive disorders of pregnancy, into a single category such as overweight‐obese, generating substantial metabolic heterogeneity across study populations (Arenas et al., 2025). Importantly, hypertensive disorders of pregnancy, such as pre‐eclampsia and gestational hypertension, have been associated with adverse lactation outcomes and alterations in breast milk composition (Arenas et al., 2025). Women with gestational hypertension and pre‐eclampsia have been reported to exhibit delayed lactogenesis, reduced milk supply, lower rates of exclusive breastfeeding and shorter lactation duration compared with normotensive women (Anna et al., 2024; Horsley et al., 2022). In addition, breast milk from mothers with gestational hypertension has been associated with increased lipid, carbohydrate and energy content (Sokołowska et al., 2023), whereas pre‐eclampsia has been linked to alterations in breast milk bioactive composition, including changes in long‐chain polyunsaturated fatty acids (PUFAs), docosahexaenoic acid, neurotrophins and other immunological and metabolic components (Dangat et al., 2014; Peila et al., 2022). In contrast, the potential impact of more severe hypertensive disorders, such as eclampsia and HELLP (haemolysis, elevated liver enzymes and low platelets) syndrome, on lactation performance, breastfeeding outcomes and breast milk composition remains poorly characterized, highlighting an important gap in the current literature. Collectively, these findings suggest that hypertensive disorders of pregnancy might independently modify both lactation physiology and breast milk composition through mechanisms involving systemic inflammation, oxidative stress, endothelial dysfunction and vascular alterations. Therefore, the coexistence of these conditions with maternal obesity might confound the interpretation of breast milk‐mediated offspring CV programming in human studies.

To assess lactational contributions independent of gestation, preclinical studies have used cross‐fostering rodent models, in which offspring born to lean or obese dams are nursed by dams of the opposite metabolic phenotype (Gorski et al., 2006; Khan et al., 2005). In these models, the postnatal environment can exert a dominant influence on offspring phenotypes, with evidence showing that the lactational environment can override prenatal influences (Gorski et al., 2006). Khan et al. (2005) applied this approach in Sprague–Dawley rats fed a high‐fat diet (HFD) and demonstrated that lactational exposure alone can exert long‐term hypertensive CV effects. Female offspring nursed by HFD‐fed dams had higher systolic blood pressure (131 ± 2.5 mmHg, *n* = 6) than control animals (119 ± 3.8  mmHg, *n* = 7, *P* < 0.05), whereas the most pronounced impairment in endothelium‐dependent relaxation of mesenteric arteries was observed in offspring exposed during lactation in both male and female offspring (*P* < 0.001; Khan et al., 2005). These findings support the concept that the lactational environment represents an important postnatal window capable of influencing long‐term CV regulation in the offspring, potentially in a sex‐specific manner.

Renal mechanisms involved in developmental programming of hypertension are well characterized in experimental models of maternal HFD exposure during gestation and lactation (Liu et al., 2023). These studies demonstrate persistent alterations in renal pathways involved in long‐term blood pressure regulation in the offspring (Inzani & Ozanne, 2022). Armitage et al. (2005, 2008) demonstrated that offspring exposed to a maternal HFD during gestation and lactation exhibit reduced renal Na^+^/K^+^‐ATPase activity together with decreased renal renin activity despite preserved kidney morphology and number of nephrons, and also showed that these alterations were particularly associated with maternal diets enriched in saturated fatty acids, supporting the concept that early‐life nutrition might influence renal sodium transport pathways involved in long‐term blood pressure regulation. Likewise, Kasper et al. (2017) demonstrated that maternal obesity during gestation and lactation induced reduced urinary sodium excretion and increased extracellular matrix deposition in the offspring, supporting the concept of long‐term renal metabolic programming. Other experimental models of maternal HFD have demonstrated increased renal AT1R expression (Kruse et al., 2017), albuminuria, glomerulosclerosis, renal fibrosis, increased markers of oxidative stress, and inflammation (Glastras et al., 2016; Larkin et al., 2021; Tain et al., 2017), supporting the involvement of renal pathways linked to long‐term blood pressure dysregulation in the offspring. However, the specific contribution of the lactation period to renal developmental programming remains poorly understood, despite the continued maturation and functional plasticity of the kidney during early postnatal life. Importantly, studies specifically targeting the lactation period have shown that maternal protein restriction can alter renal Na^+^ transport and local angiotensin II signalling pathways in adult offspring, supporting the concept that the lactational environment itself might contribute to long‐term renal regulation of blood pressure (Luzardo et al., 2011). These findings suggest that renal sodium transport and inflammatory/oxidative renal pathways might represent plausible downstream mechanisms through which obesity‐associated alterations during lactation could contribute to offspring CV risk.

Together, these findings indicate that the lactational period represents an independent and highly plastic window for offspring CV programming. However, small sample sizes and species‐specific physiology limit direct extrapolation to humans, and the precise contribution of obesity‐associated alterations in breast milk components to these long‐term CV outcomes remains unknown. Nevertheless, these alterations represent plausible biological pathways through which maternal obesity during lactation might influence postnatal developmental programming (Table 1).

**TABLE 1:** Key studies evaluating maternal obesity‐associated changes in breast milk composition and their potential implications for offspring cardiovascular programming.

Studies in patients or human samples					
Study design	Maternal phenotype	Lactation stage	Main findings	Proposed offspring outcome	Reference
Prospective cohort follow‐up (*n* = 56)	Pre‐pregnancy BMI ≤ 25 kg/m^2^ (*n* = 34) vs. BMI > 25 kg/m^2^ (*n* = 22). GWG also assessed	Colostrum, 1 and 6 months postpartum	↓ TGF‐β2, sCD14 and IL‐6. Altered milk microbiota	Impaired immune maturation, dysbiosis, and low‐grade inflammation	Collado et al. (2012)
Secondary analysis of prospective mother–infant cohort (*n* = 40)	Postpartum BMI < 30 kg/m^2^ (*n* = 20) vs. BMI ≥ 30 kg/m^2^ (*n* = 20)	1 and 4 months postpartum	↑ milk leptin and n‐6:n‐3 PUFA ratio. IL‐8, IL‐6 and IL‐1β linked to ↑ infant weight gain and BMI *z*‐scores	Early adiposity, low‐grade inflammation, and later hypertensive susceptibility	Enstad et al. (2021)
Cross‐sectional study (*n* = 68)	Pre‐pregnancy BMI 18.5–24.9 kg/m^2^ (*n* = 25) vs. 25.0–29.9 kg/m^2^ (*n* = 24), vs ≥30 kg/m^2^ (*n* = 19)	Colostrum	Obesity was associated with ↑ colostrum glucose, calories and fat. Overweight/obesity showed ↑ colostrum sIgA	Altered neonatal metabolism, immune maturation, adiposity, and later CV risk	Fujimori et al. (2015)
Observational study (*n* = 65)	Pre‐pregnancy BMI < 25 kg/m^2^ (*n* = 47) vs ≥30 kg/m^2^ (*n* = 18)	≤6 months postpartum	Differential expression of 19 EV‐derived microRNAs. Target pathways include FoxO, EGFR/ErbB and prolactin signalling	Altered gene expression, immune regulation, and metabolic pathways linked to CV risk	Cho et al. (2022)

Studies in experimental animals					
Species/Strain	Dietary fat (%)	Intervention period	Age at measurement	Main findings	Reference
Rat/Sprague–Dawley	25.7	Pregnancy and/or suckling	180 days	Maternal HFD ↑ adult offspring blood pressure. Lactational exposure induced endothelial dysfunction, with ↑ adiposity and hyperinsulinaemia	Khan et al. (2005)
Rat/Sprague–Dawley	32	Pregnancy and/or suckling	15 weeks	↑ Milk insulin and altered fatty acid composition, associated with ↑ adiposity and insulin resistance in offspring	Gorski et al. (2006)

Abbreviations: BMI, body mass index; CV, cardiovascular; EV, extracellular vesicle; GWG, gestational weight gain; HFD, high‐fat diet; PUFA, polyunsaturated fatty acid.

### 4.1 Immune mediators and microbiota in breast milk and offspring hypertensive CV programming

Breast milk contains a complex and dynamic network of immune mediators and microbial components that actively shape early‐life immune maturation during a critical window of CV development (de Groen et al., 2026). Maternal obesity promotes inflammatory remodelling of the mammary gland microenvironment, characterized by elevated local cytokine and adipokine signalling, increased macrophage infiltration within mammary adipose tissue and the formation of crown‐like structures surrounding hypertrophic or dying adipocytes (Vaysse et al., 2017). These alterations might influence the immune composition of breast milk. Given the well‐established role of chronic low‐grade inflammation in endothelial dysfunction and hypertension (Zhang et al., 2023), obesity‐associated changes in the breast milk immune profile represent biologically plausible mechanisms linking maternal obesity during lactation to offspring hypertensive CV programming.

Studies evaluating cytokine concentrations in breast milk from mothers with elevated BMI have reported heterogeneous findings. In a prospective cohort of 56 mother–infant dyads (34 with pregestational BMI ≤ 25 kg/m^2^ and 22 with BMI > 25 kg/m^2^), immune mediators were assessed in colostrum and mature milk at 1 and 6 months of lactation (Collado et al., 2012). Mothers with BMI > 25 kg/m^2^ exhibited lower concentrations of transforming growth factor (TGF)‐β2, soluble CD14 and interleukin(IL)‐6 in milk collected at 1 month postpartum (Collado et al., 2012). Collectively, this suggests not simply a shift towards increased or decreased inflammation, but rather a dysregulated immunomodulatory profile in the breast milk associated with maternal BMI. Such alterations might influence early immune tolerance, microbial sensing and host–microbe interactions during a critical developmental window (Bermejo‐Haro et al., 2023; Collado et al., 2012; Na et al., 2023; Wang et al., 2023). However, the relatively small sample size and the inclusion of women with BMI > 25 kg/m^2^ as a single category limit the ability to distinguish overweight‐ from obesity‐specific effects (Collado et al., 2012). Nevertheless, these findings provide mechanistic plausibility for the hypothesis that maternal obesity might transmit an altered immunological signature through breast milk during a sensitive period of vascular and metabolic maturation.

In another analysis of 40 mother–infant dyads (20 mothers with BMI < 30 kg/m^2^ and 20 with BMI ≥ 30 kg/m^2^), breast milk samples collected at 1 and 4 months postpartum were analysed for inflammatory mediators (Enstad et al., 2021). In this cohort, maternal BMI ≥ 30 kg/m^2^ was not associated with significant differences in breast milk concentrations of IL‐8, IL‐6 or IL‐1β (Enstad et al., 2021). However, longitudinal analyses revealed that greater exposure to breast milk IL‐8 and IL‐6 was positively associated with accelerated infant weight gain and increased BMI during early life (Enstad et al., 2021). These findings suggest that variability in early cytokine exposure might still influence postnatal growth trajectories. Importantly, this study classified women based on postpartum BMI without clearly distinguishing pregestational obesity from EGWG, which might reflect distinct metabolic and inflammatory phenotypes. This limitation underscores the heterogeneity frequently observed across human studies evaluating immune mediators in breast milk, where differences in maternal metabolic characterization, sampling timing and lactation stage complicate direct comparisons. Moreover, human cohorts are inherently influenced by multiple confounders that might independently modulate breast milk immune composition and infant growth outcomes. Given the established role of early‐life inflammation in endothelial dysfunction, vascular remodelling, altered nitric oxide (NO) bioavailability and long‐term hypertension risk, such alterations in breast milk‐derived cytokine exposure might represent an indirect pathway contributing to inflammatory vascular priming in early life.

At the cellular level, cross‐sectional studies have reported phenotypic differences in leucocyte subpopulations between peripheral blood and colostrum of women with obesity, including a reduced proportion of B lymphocytes, although their activation state and functional role remain poorly characterized (Piñeiro‐Salvador et al., 2022). Given that B lymphocytes are the precursors of antibody‐secreting plasma cells and key contributors to regulatory immune signalling, reduced transfer of B lymphocytes through the breast milk could therefore alter early immune education, potentially favouring a pro‐inflammatory phenotype and reducing mechanisms that normally constrain vascular oxidative stress and endothelial activation (Gururajan et al., 2014). Consistent with this axis, maternal obesity has also been associated with changes in Ig content. Some studies report increased soluble IgA in colostrum with largely preserved IgG and IgM levels (Fujimori et al., 2015), whereas others have observed lower concentrations of IgG, IgA and IgM across the postpartum period in women with obesity in comparison to control women of normal weight, with reductions approaching 2‐fold and progressively worsening with increasing BMI (Dyndar et al., 2019). Reduced Ig concentrations might weaken passive mucosal immunity, compromise gut barrier integrity and disrupt host–microbiota interactions, thereby promoting dysbiosis and low‐grade inflammation (Froń & Orczyk‐Pawiłowicz, 2023). Together, these findings suggest that maternal adiposity might influence both the cellular and humoral arms of breast milk immunity. Altered transfer of immune cells and antibodies during this developmental window could modify mucosal immune education, host–microbiota interactions and systemic inflammatory tone, thereby contributing to sustained endothelial activation, enhanced vascular reactivity and increased susceptibility to hypertensive stimuli later in the infant's life.

In addition to direct immune signalling, breast milk contains a diverse microbial community that contributes to early‐life gut colonization, with *Staphylococcus* spp., *Streptococcus* spp. and lactic acid bacteria among the most frequently detected taxa (Zimmermann & Curtis, 2020). Commensal bacteria transferred through milk might seed the infant gut and interact with immune and epithelial cells during a critical window of intestinal and systemic maturation (de Groen et al., 2026). High BMI has been associated with reduced microbial diversity and altered taxonomic composition in colostrum and mature breast milk, including lower relative abundance of *Bifidobacterium* and higher representation of *Staphylococcus* species (Collado et al., 2012; Zimmermann & Curtis, 2020). Early‐life dysbiosis has been linked to increased intestinal permeability and enhanced translocation of microbially derived components, such as lipopolysaccharide, into the systemic circulation, which activates Toll‐like receptor signalling and modulates miRs affecting host gene expression (Mousa et al., 2022). This interaction influences lymphocyte differentiation, cytokine production, epithelial turnover, gut barrier integrity and autophagy, promoting low‐grade systemic inflammation, oxidative stress and endothelial activation (Mousa et al., 2022). Thus, obesity‐associated alterations in breast milk microbiota might contribute indirectly to vascular developmental programming. Although direct evidence linking immune components of breast milk to overt offspring CV disease remains limited, these mechanisms position breast milk immune alterations as potential upstream contributors to inflammatory vascular priming and heightened hypertension susceptibility later in life.

### 4.2 Hormonal and metabolic signals in breast milk and their potential role in offspring hypertensive CV risk

Among the immune mediators altered in breast milk, metabolic hormones represent a key pathway through which maternal obesity might influence offspring cardiometabolic development. Maternal obesity is associated with alterations in the hormonal and metabolic composition of breast milk, particularly affecting adipokines and appetite‐regulating hormones. Several studies report that mature breast milk from mothers with obesity contains higher concentrations of leptin, adiponectin, ghrelin, insulin and obestatin, in addition to higher levels of total fat, glucose and lactoferrin (Fujimori et al., 2015; Tekin Guler et al., 2022). These alterations have been described in cohorts characterized by elevated pre‐pregnancy BMI and EGWG; however, heterogeneity in study design, population characteristics and obesity phenotyping is likely to contribute to the variability across reports. Collectively, this evidence indicates that maternal metabolic status during lactation influences the endocrine profile of breast milk.

Among these components, adipokines, such as leptin, adiponectin and ghrelin, play central roles in the regulation of appetite, adiposity, insulin sensitivity and energy homeostasis (Samuelsson et al., 2013; Taylor et al., 2014). Interestingly, some studies suggest that the relationship between maternal BMI and breast milk hormonal composition might differ according to offspring sex. Fields et al. (2017) reported that insulin and leptin concentrations in human milk were highest in mothers of female infants at very high maternal BMI, suggesting potential sex‐specific adaptations in obesity‐associated milk signalling. Leptin, consistently reported to be elevated in the breast milk of mothers with obesity, is a key developmental signal involved in the formation of hypothalamic circuits that regulate appetite and energy balance (Froń et al., 2025; Obradovic et al., 2021). Experimental studies suggest that early‐life exposure to altered leptin levels might modify hypothalamic leptin sensitivity, potentially predisposing offspring to persistent hyperphagia, increased adiposity and leptin resistance (Alexe et al., 2006). Importantly, leptin is also a regulator of sympathetic nervous system activity and renal sodium handling, both of which are central determinants of long‐term blood pressure control. Chronic hyperleptinaemia has been implicated in sympathetic overactivation, endothelial dysfunction and impaired NO bioavailability.

In contrast to the anorexigenic actions of leptin, the endocannabinoid (EC) system acts as a complementary neurohormonal regulator of appetite, energy balance and CV homeostasis (Pagano et al., 2008; Silver, 2019). EC stimulates food intake and promotes energy storage, but chronic EC system overactivation (commonly observed in obesity), contributes to metabolic inflammation and cardiometabolic dysregulation (Schulz et al., 2021). The principal cannabinoid receptors (CB_1_ and CB_2_) are expressed in mammary tissue, where they participate in inflammatory signalling and adipose tissue remodelling (Elgueta et al., 2026). ECs such as 2‐arachidonoylglycerol (2‐AG) and anandamide (AEA) have been detected in breast milk, and evidence suggests that maternal BMI and EGWG might influence their concentrations, with higher 2‐AG levels reported in mature breast milk of women with overweight and obesity (Datta et al., 2021; Gaitán et al., 2018; Pontes et al., 2025). Nonetheless, the physiological role of breast milk‐derived EC remains incompletely characterized. Increased early‐life exposure to these signals might influence hypothalamic appetite regulation, energy balance and inflammatory tone. Moreover, the endocannabinoid system acts as a modulator of CV function by reducing cardiac contractility and promoting vasodilatation, particularly in hypertensive conditions (Bátkai et al., 2004). Together, these observations suggest that obesity‐associated alterations in milk EC signalling might represent an additional neurohormonal pathway linking maternal metabolic status during lactation with offspring cardiometabolic regulation and long‐term hypertensive CV risk.

Beyond their metabolic actions, adipokines and related hormonal signals also regulate vascular tone, endothelial function and sympathetic nervous system activity (Hemat Jouy et al., 2024). Accordingly, early postnatal exposure to altered levels of these bioactive milk components might influence vascular and renal pathways involved in blood pressure regulation, providing a plausible mechanistic link between maternal obesity during lactation and increased hypertensive CV risk later in life. Nevertheless, direct evidence specifically linking breast milk‐derived adipokines to development of offspring CV disease remains limited.

### 4.3 Breast milk lipid composition in maternal obesity and implications for offspring hypertensive CV risk

Alterations in the lipid composition of breast milk represent an additional pathway through which maternal obesity might influence metabolic and vascular programming in the offspring. Maternal obesity has been associated consistently with alterations in the lipid composition of breast milk, particularly affecting total fat content and the profile of PUFAs (Enstad et al., 2021; Panagos et al., 2016). Several studies in women with obesity, overweight or EGWG report higher total lipid concentrations in breast milk, together with an increased ratio of pro‐inflammatory omega‐6 (n‐6) to omega‐3 (n‐3) PUFAs (Enstad et al., 2021; Panagos et al., 2016). This shift towards an n‐6‐enriched profile has been linked to concurrent changes in other metabolic and inflammatory milk components, including insulin, C‐reactive protein, leptin, adiponectin, ghrelin, IL‐6 and tumor necrosis factor‐α, suggesting coordinated alterations in the bioactive milieu delivered to the infant (Álvarez et al., 2020; Panagos et al., 2016)

From a mechanistic perspective, elevated exposure to n‐6 PUFAs might alter the eicosanoid cascade, favouring the production of arachidonic acid‐derived metabolites, such as prostaglandins, that promote inflammatory signalling, vascular reactivity and endothelial activation (Drake & Reynolds, 2010; Tekin Guler et al., 2022). In contrast, n‐3 PUFAs exert anti‐inflammatory and vasoprotective effects, enhancing NO bioavailability and limiting oxidative stress (Oppedisano et al., 2020). Experimental studies support this concept. Maternal exposure to an HFD during pregnancy and lactation increases n‐6 PUFA levels in breast milk and the neonatal circulation, leading to persistent alterations in offspring lipid metabolism, hepatic triglyceride accumulation, insulin resistance and inflammatory signalling (Álvarez et al., 2020). In rodents, these changes are accompanied by impaired mitochondrial β‐oxidation, increased *de novo* lipogenesis and sustained modifications in gene expression related to adipocyte differentiation, lipid turnover and inflammatory pathways (Álvarez et al., 2020). Importantly, such metabolic disturbances are closely linked to endothelial dysfunction, increased oxidative stress and heightened vascular sensitivity to hypertensive stimuli later in life (Tran et al., 2020).

Clinically, higher breast milk n‐6:n‐3 PUFA ratios and elevated lipid‐associated hormones in mothers with obesity have been associated with accelerated infant weight gain (Howie et al., 2009; Wijenayake et al., 2023), a recognized early risk factor for later cardiometabolic and hypertensive risk. Although direct evidence linking obesity‐associated alterations in breast milk lipid composition to offspring hypertensive CV disease remains limited, these findings support a plausible role for lipid‐mediated postnatal programming mechanisms that might contribute to long‐term vascular dysfunction, increased arterial stiffness and susceptibility to elevated blood pressure.

### 4.4 Breast milk EVs as mediators of postnatal hypertensive CV programming

More recently, breast milk‐derived EVs have emerged as an additional layer of complexity in lactational signalling, carrying bioactive molecules that might modulate gene expression and influence endothelial and inflammatory pathways relevant to CV development in the offspring. In vitro studies demonstrate that EVs isolated from healthy human breast milk attenuate lipopolysaccharide‐induced endothelial activation, reducing IL‐6 and VCAM‐1 expression, inhibiting nuclear factor‐κB signalling and limiting mitochondrial oxidative stress (Cho et al., 2025). *Ex vivo* studies also demonstrate that treatment with human milk EVs from healthy mothers restores endothelium‐dependent vasorelaxation in mesenteric arteries from diet‐induced obese mice, supporting a functional impact on vascular reactivity (Cho et al., 2025). These findings provide mechanistic evidence that breast milk EVs can directly influence endothelial function and vascular reactivity during the early life of offspring.

The biological activity of breast milk EVs is mediated by their molecular cargo, including miRs, long non‐coding RNAs, proteins and lipids, which can modulate gene expression and metabolic signalling pathways (Jiang et al., 2021). Experimental studies demonstrate that breast milk EVs can influence T‐cell development, promote tolerogenic responses and attenuate excessive inflammatory activation (Vahkal et al., 2025; Zonneveld et al., 2021). Several breast milk EV miRs, such as miR‐148a, miR‐30b, miR‐222 and members of the let‐7 family, regulate pathways involved in immune regulation, endothelial function and metabolic control, which are processes central to long‐term CV health (Herwijnen et al., 2018; Zeng et al., 2021).

Maternal metabolic status appears to influence the characteristics and cargo of breast milk EVs (Chondrogianni et al., 2025). Although some studies have examined concentrations of EVs in relationship to maternal obesity, the findings have been inconsistent, with some reporting altered numbers of EVs and others finding no significant differences (Cho et al., 2021). These discrepancies might reflect methodological variations in the isolation and quantification of EVs, in addition to differences in the studied populations and timing of milk collection (Hu et al., 2021). The most extensively documented obesity‐associated changes in breast milk EVs involve profiles of miRs. Several studies report differential expressions of miRs in breast milk EVs from mothers with obesity compared with normal‐weight mothers (Cho et al., 2022; Chondrogianni et al., 2025). In a comprehensive study of 65 nursing mothers, 19 differentially expressed miRs were identified in breast milk EVs from mothers with pre‐pregnancy obesity (BMI ≥ 30 kg/m^2^), including miR‐575, miR‐642a‐3p and miR‐652‐5p (Cho et al., 2022). Bioinformatic analyses indicated enrichment of target pathways related to FoxO signalling, EGFR/ErbB signalling and metabolic regulation (Cho et al., 2022). These pathways are critically involved in the oxidative stress responses, endothelial survival, vascular remodelling and insulin signalling, suggesting that obesity‐associated alterations in the miR cargo of EVs might influence molecular networks that regulate cardiometabolic development in the offspring (Ferber et al., 2012; Zhou et al., 2021).

Other miRs in breast milk EVs, such as miR‐148a and miR‐30b, are downregulated in mothers with overweight and obesity (Shah et al., 2021). These EVs are implicated insulin signalling, lipid metabolism, macrophage activation and adipogenesis, suggesting potential links to cardiometabolic programming (Shah et al., 2021; Szeliga et al., 2025). In animal models, maternal exposure to a HFD and supplementation with milk‐derived exosomes (a small endosomal subtype of EVs) have been associated with altered DNA methyltransferase 1 expression and persistent changes in DNA methylation patterns in offspring tissues, supporting an epigenetic mechanism by which the cargo of EVs might influence long‐term metabolic and CV risk (Eisha, 2021).

Collectively, breast milk EVs represent a biologically active component of breast milk that can modulate immune, metabolic and vascular pathways during lactation. Emerging evidence indicates that maternal obesity alters its composition, particularly its miR cargo, raising the possibility that EV‐mediated signalling contributes to postnatal CV programming. Although direct longitudinal evidence linking breast milk EV profiles to offspring hypertension or overt CV disease remains limited, the convergence of epigenetic regulation, endothelial modulation, inflammatory signalling and metabolic control provides a physiologically plausible framework. By influencing gene networks during endothelial maturation and sympathetic regulation, breast milk EVs might function as upstream modulators of long‐term CV susceptibility, particularly in the offspring of obese mothers.

**FIGURE 2:**
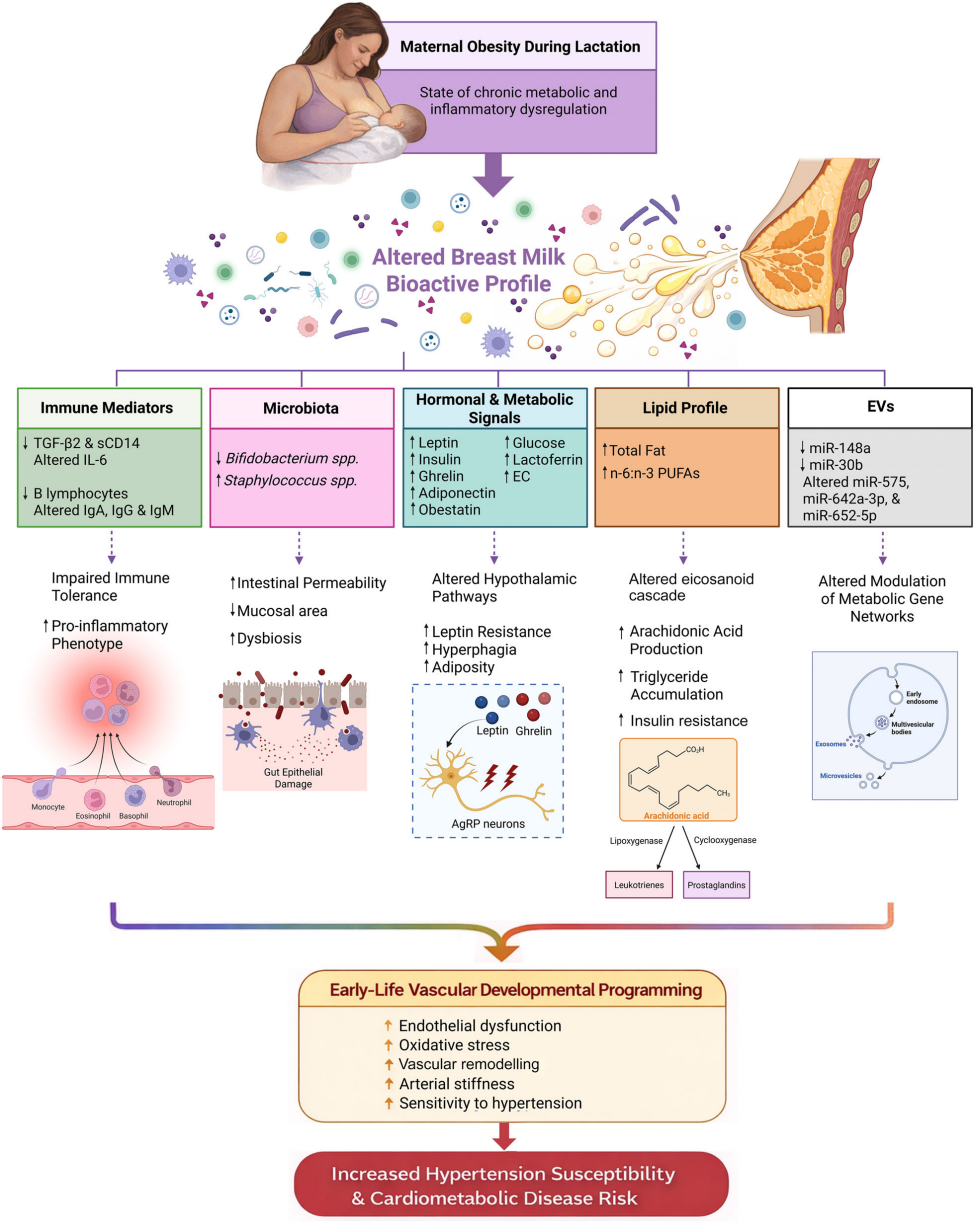
Proposed breast milk‐mediated pathways linking maternal obesity during lactation to postnatal cardiovascular programming. Maternal obesity is associated with a chronic metabolic and inflammatory milieu that might alter the bioactive composition of breast milk. Exposure to immune, hormonal, lipid and vesicular signals during early postnatal life (a period of high developmental plasticity) might converge on key cardiovascular pathways. Dysregulation of these pathways might contribute to endothelial dysfunction, altered vascular reactivity and long‐term blood pressure regulation in the offspring. Imagen created with BioRender.com. Abbreviations: AgRP: agouti‐related peptide; EC: endocannabinoid; PUFAs: polyunsaturate fatty acids; EVs: extracellular vesicles; IgA: immunoglobulin A; IgG: immunoglobulin G; IgM: immunoglobulin M; IL‐6: interleukin‐6; miR: microRNA; n‐3: omega‐3 fatty acids; n‐6: omega‐6 fatty acids; sCD14: soluble cluster of differentiation 14; TGFβ‐2: transforming growth factor beta 2.

Taken together, the evidence reviewed suggests that maternal obesity induces coordinated alterations across multiple components of breast milk, including inflammatory mediators, hormones, lipids, microbiota and EVs. Rather than acting in isolation, these factors might converge on shared biological pathways involved in vascular function, metabolic regulation and renal development, ultimately shaping offspring hypertensive CV risk (Figure 2).

## 5 FUTURE DIRECTIONS

Despite growing evidence that maternal obesity alters the immunological, hormonal, lipid and EV composition of breast milk, direct links between these alterations and long‐term offspring CV outcomes remain insufficiently characterized. Most available human studies are observational, involve relatively small cohorts, and focus predominantly on early growth or metabolic parameters rather than vascular function or blood pressure trajectories. Future research should prioritize longitudinal studies integrating rigorous maternal metabolic phenotyping and clearly distinguishing pregestational obesity, EGWG and overweight. Consequently, the contribution of altered breast milk‐derived bioactive factors to sustained CV programming remains largely inferential.

Mechanistic investigations are also needed to clarify whether breast milk‐derived immune mediators, cells, adipokines, lipid composition and EV cargo directly influence pathways involved in NO bioavailability, oxidative stress balance and sympathetic nervous system regulation. Experimental models might help to disentangle prenatal from postnatal influences and determine whether exposure during lactation sensitizes offspring to hypertensive stimuli later in life. Additionally, potential sex‐specific responses to obesity‐associated alterations in breast milk composition warrant further investigation, because emerging data suggest differential susceptibility to hypertensive CV programming between males and females. Incorporating sex as a biological variable might refine risk prediction and reveal mechanistic divergence in vascular adaptation.

Understanding the potential consequences in children exposed to nutritional and metabolic perturbations during critical windows of development is essential for designing early interventions aimed at reducing the risk of obesity, metabolic dysfunction and CV disease later in life. Lactation represents a key postnatal window during which immune, neuroendocrine and epigenetic signals conveyed through breast milk might contribute to the modulation of vascular maturation, endothelial function and neurohumoral regulation. However, further well‐designed longitudinal and mechanistic studies are required to establish causal relationships and to define more precisely the contribution of lactation to offspring CV risk.

## AUTHOR CONTRIBUTIONS

Gabriela Arenas wrote the manuscript and developed the full text. Cristián A. Amador and Susana Contreras‐Duarte conceived the review idea and provided in‐depth critical revision of the manuscript. Cristián A. Amador and Susana Contreras‐Duarte reviewed the final version of the text. All authors read and approved the final manuscript and agree to be accountable for all aspects of the work in ensuring that questions related to the accuracy or integrity of any part of the work are appropriately investigated and resolved. All persons designated as authors qualify for authorship, and all those who qualify for authorship are listed.

## CONFLICT OF INTEREST

The authors declare no conflicts of interest.
